# Anti-Colorectal Cancer Activity of *Panax* and Its Active Components, Ginsenosides: A Review

**DOI:** 10.3390/ijms26062593

**Published:** 2025-03-13

**Authors:** Han Su Kang, Hyun Kyung Lim, Won Young Jang, Jae Youl Cho

**Affiliations:** Department of Integrative Biotechnology, Sungkyunkwan University, Suwon 16419, Republic of Korea; ssu2010@naver.com (H.S.K.); lhk1091605@naver.com (H.K.L.); wybest0327@naver.com (W.Y.J.)

**Keywords:** colorectal cancer, genus *Panax*, ginsenosides, anti-cancer, drug delivery

## Abstract

Colorectal cancer (CRC) poses a significant health burden worldwide and necessitates novel treatment approaches with fewer side effects than conventional chemotherapy. Many natural compounds have been tested as possible cancer treatments. Plants in the genus *Panax* have been widely studied due to their therapeutic potential for various diseases such as inflammatory disorders and cancers. Extracts from plants of genus *Panax* activate upstream signals, including those related to autophagy and the generation of reactive oxygen species, to induce intrinsic apoptosis in CRC cells. The root extract of *Panax notoginseng* (*P. notoginseng*) regulated the gut microbiota to enhance the T-cell-induced immune response against CRC. Protopanaxadiol (PPD)-type ginsenosides, especially Rh2, Rg3, Rb1, and Rb2, significantly reduced proliferation of CRC cells and tumor size in a xenograft mouse model, as well as targeting programmed death (PD)-1 to block the immune checkpoint of CRC cells. Moreover, modified nanocarriers with ginsenosides upregulated drug efficacy, showing that ginsenosides can also be utilized as drug carriers. An increasing body of studies has demonstrated the potential of the genus *Panax* in curing CRC. Ginsenosides are promising active compounds in the genus *Panax*, which can also support the activity of conventional cancer therapies.

## 1. Introduction

Colorectal cancer (CRC) has the third-highest incidence and mortality among cancers worldwide [1]. CRC can occur through three different mechanisms, namely the CpG island methylator phenotype (CIMP), chromosomal instability (CIN), and microsatellite instability (MSI) [2]. CpG island hypermethylation induces the transcriptional deactivation of tumor suppressor genes [3]. The CIN pathway results in the mutation of *KRAS*, *TP53* and *APC*, which are oncogene and tumor suppressor genes, respectively [4,5]. MSI is driven by an impaired DNA mismatch repair system. In total, 15% of CRC patients show MSI and, in these cases, conventional chemotherapies do not work well [6]. Drug resistance related to MSI makes it hard to cure CRC [7]. The high mortality rate of CRC is also attributed to its ability to metastasize well, especially in the peritoneum and liver [8]. Synchronous metastasis is most common in the liver in approximately 20% of patients, and metachronous metastasis occurs within 5 years in up to 60% of patients [9]. Patients with poor prognosis often exert metastasis in the peritoneum.

In the early stages of CRC, tumors can be removed via surgical procedures [10]. However, patients who are not candidates for surgery should undergo chemotherapy or radiotherapy to reduce the size of the tumor. The most well-known first-line chemotherapy drug is fluoropyrimidine (5-FU) [11]. 5-FU is a uracil analog where the hydrogen in the C-5 position is replaced by fluorine [12]. 5-FU can be converted to active metabolites such as fluorouridine triphosphate (FUTP), which blocks nucleotide synthetic enzyme thymidylate synthase (TS) and RNA synthesis in cancer cells [13]. 5-FU can be used with other drugs that induce DNA damage, such as oxaliplatin (OX) or irinotecan (IRI), to maximize cytotoxicity; or folinic acid, which can counteract the toxic effect of chemotherapy on patients. These combined therapies can be defined as FOLFOX (5-FU + folinic acid + OX), FOLFIRI (5-FU + folinic acid + IRI), or FOLFOXIRI (5-FU + folinic acid + OX + IRI) [14,15,16]. Unfortunately, the side effects of these therapies, including low tumor-specific selectivity and systemic toxicity, interfere with the recovery of the patient’s health [17]. Cancer cachexia accompanying body weight loss and muscle weakness is a major side effect of chemotherapy [18,19]. Therefore, extensive research has been conducted to develop CRC treatments with reduced side effects [20].

*Panax* is a genus of perennial herbs in Asia and North America with tuberous roots, verticillate leaves, and a solitary umbel of flowers [21,22,23,24,25,26]. The three major *Panax* species are *Panax ginseng* (*P. ginseng*) Meyer, *Panax notoginseng* (*P. notoginseng*) FH Chen, and *Panax quinquefolium* L. (*P. quinquefolium* L.) [22,27]. The pharmacological activities of the roots of each plant have been extensively investigated [28,29,30,31,32,33]. The root extracts of these plants can alleviate symptoms of inflammatory disorders (e.g., lung injury), colitis, gastritis, and nephropathy as well as cancer [34,35,36,37]. The root extract of *P. notoginseng* inhibited 4T1 breast cancer cell survival and proliferation [38]. *P. quinquefolius* extract also exerted an anti-tumor effect on the DU145 prostate cancer cell line via the activation of an intrinsic apoptotic pathway [39]. *P. ginseng*—probably the most studied herbal plant worldwide—has been reported to induce apoptosis and the cell cycle arrest of cancer cells [40,41]. *P. ginseng* extracts also inhibited angiogenesis and endothelial–mesenchymal transition (EMT), suppressing metastasis [40,41,42,43,44,45,46]. Recently, *P. ginseng* berry extract has also attracted attention from researchers. *P. ginseng* berry-derived polysaccharides can activate NK cells while deactivating regulatory T cells to moderate the tumor immune environment [47,48].

**Figure 1:**
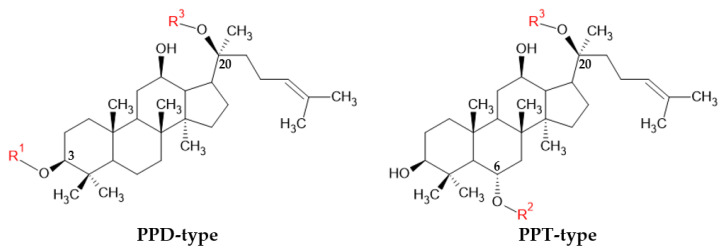
Chemical structures of PPD- and PPT-type ginsenosides.

Most of the active compounds in the genus *Panax* are ginsenosides, which are triterpene saponins [28,44,49,50,51,52,53]. Ginsenosides can be classified as protopanaxadiol (PPD)-type ginsenosides and protopanaxatriol (PPT)-type ginsenosides [54]. PPD and PPT both have R groups, which may be substituted with glucose (Figure 1). In PPD-type ginsenosides, either C-3 or C-20 carbon can be linked with glucose, while C-6 or C-20 carbon can be attached to glucose in PPT-type ginsenosides [41,55]. Rp1 is also a PPD-type ginsenoside but can be prepared via the reduction of other ginsenosides like Rg3 and Rk1 with hydrogeneration [56]. Interestingly, recent studies on the anti-cancer effect of ginsenosides have mainly focused on PPD-type ginsenosides such as Rh2, Rg3, Rb1, Rb2, Rp1, and compound K (CK) (Table 1). Du et al. also revealed that PPD and its derivatives exert antiproliferative activities on the HCT-116 colorectal cancer cell line, SW-480 colorectal cancer cell line, and MCF-7 breast cancer cell line [57]. Our previous study also revealed that 20(S)-PPD from *P. ginseng* targeted gastric cancer cells by inducing autophagy [58].

Drug delivery systems have been rapidly developed, as the tumor microenvironment is a significant hurdle for cancer-selective treatment [59,60]. Efforts to deliver ginsenosides with nanocarriers, including micelles, liposomes, and gold nanoparticles (NPs), have also been undertaken to enhance their bioavailability and therapeutic efficacy [61]. Wu et al. revealed that Rg3-NPs supplemented with modified chitosan strengthened the anti-cancer effect of doxorubicin [62]. Compound K encapsulated in liposome was able to circulate in the body for long periods of time and actively target tumors in xenograft mouse models [63]. Moreover, ginsenosides themselves can be utilized as drug carriers, as they mimic the cholesterol structure in nano-lipid particles. Rg3-based liposomes improve selectivity for cancer cells through recognizing the GLUT receptor on the tumor cell membrane [64]. A long-lasting effect can also be expected as Rh2 can interrupt the phagocytic activity of monocytes, which can remove drugs before acting on the lesion [65].

In this review, we organize recent studies evaluating the therapeutic potential of the genus *Panax* and its active compounds—ginsenosides—on CRC. To narrow down the topic, we focused on articles published since 2019 using PubMed, Embase Web of Science, and Scopus. We divided the content into three sections covering the efficacies of plant extracts and ginsenosides and the role of ginsenosides as a drug carrier. As CRC is still a deadly disease and there is a high demand for its treatment, we hope our research will be an opportunity to shed light on the development of CRC treatments. The graphical abstract was drawn utilizing BioRender.com, while the chemical structures of ginsenoside and derivatives were drawn using ACD/ChemSketch version 2023.1.1 (Advanced Chemistry Development, Inc., Toronto, ON, Canada).

## 2. Studies of the Mechanism of Colon Cancer Treatment of the Genus *Panax*

Natural products are crucial sources of drug candidates targeting cancer [66]. They exert anti-cancer activity through reducing the cell viability and proliferation of cancer cells [67]. Most of them target mitochondrial membrane damage via inducing an intrinsic apoptotic pathway. The intrinsic apoptotic pathway is controlled by the balance between the anti-apoptotic Bcl-2 family and pro-apoptotic B-cell lymphoma-2 (Bcl-2) family. Bcl-2, Bcl-XL, and Bcl-W represent the anti-apoptotic Bcl-2 family, while the pro-apoptotic Bcl-2 family includes Bcl-2-associated X protein (Bax), Bak, and Bad [68,69]. The Bcl-2/Bax ratio is reduced as upstream molecules, such as p53 and cytochrome c, are released from mitochondria, leading to cleavage of the caspase family [70]. The cleaved caspase promotes the degradation of cytoplasmic substrates and DNA fragmentation, which eventually leads to apoptosis [71].

### 2.1. Apoptosis Pathway

**Figure 2:**
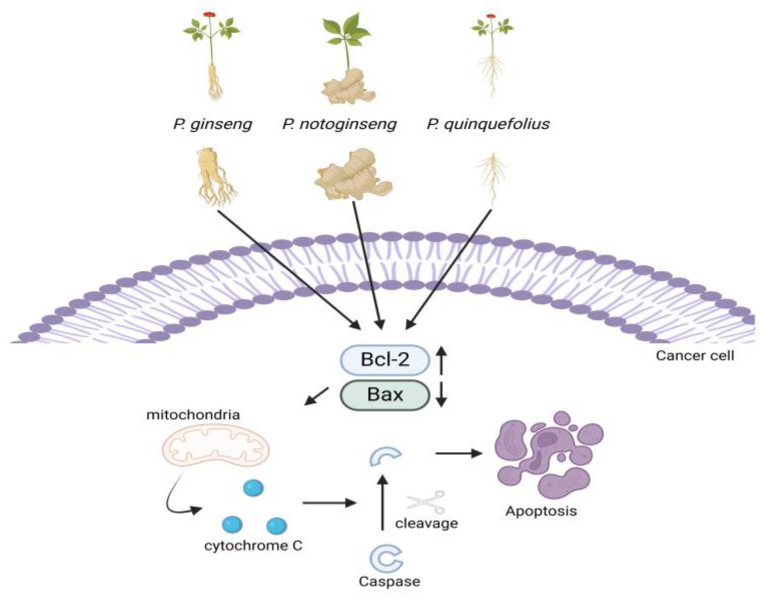
The anti-apoptotic activity of the genus *Panax* plants targeting cancer cells.

Interestingly, extracts of the genus *Panax* also upregulate the intrinsic apoptotic pathway to target cancer cells (Figure 2) [41]. For instance, 600 μg/mL of *P. notonsingeng* reduced the viability of Y79 human retinoblastoma cells up to 50% via increasing the expression of PTEN, which acts as an antagonist of the PI3K/Akt pathway [72]. As PI3K/Akt blocks the role of BAX, the extract recovers the activity of BAX and induces apoptosis in Y79 cells [73]. Jeong et al. also suggested that *P. ginseng* water extract induces cleavage of caspases 3 and 9 [74]. The effect is driven by the p53-independent activation of BAX. The extract triggered Noxa activation accompanied by endoplasmic reticulum (ER) stress in cancer cells. Recent studies on the CRC treatments with the genus *Panax* extract also focused on the role of the extract as an apoptosis inducer. Wang et al. showed that *P. ginseng* berry ethanol extract significantly reduced the cell viability of HCT-116 and HT-29 human CRC cells [75]. In fact, the extract dose-dependently induced both early and late apoptosis, which was detected with Annexin V staining. The extract (300 μg/mL) upregulated the mRNA expression of pro-apoptotic agents such as p53, Bad, Bax, caspase 3, 8, and 9 while downregulating the mRNA expression of anti-apoptotic agents such as Bcl-2. Meanwhile, the root extract of *P. ginseng* generated reactive oxygen species (ROS) in the HCT-116 (at 2 mg/mL) and SNU-1033 (at 2.3 mg/mL) CRC cell lines [76]. This eventually induced the cleavage of caspases 3 and 9, triggering the apoptotic degradation of the cells. The hairy roots from *P. quinquefolius* exerted cytotoxicity on Caco-2 CRC cells [77]. Specifically, the root cultures elicited methyl jasmonate for seven days to promote synthesis of ginsenosides, and it has been reported that methyl jasmonate enhances the synthesis of PPD-type ginsenosides via an activating enzyme called 3-hydroxy-3-methylglutaryl-CoA reductase [78]. This process enhanced the extract’s ability to induce intracellular ROS production in Caco-2 cells.

### 2.2. Autophagy

Autophagy is a process of recycling intracellular organelles via autophagosomes. The organelles packed in autophagosomes can be further degraded in the lysosome and used as a source of new amino acids, nucleotides, and fatty acids. Autophagy is involved in various physiological effects like innate immune systems, cell death, aging, and cancer. Our previous study also showed that the *P. ginseng* berry harbors syringaresinol, a plant lignan that induces autophagy in keratinocytes to prevent skin aging [79]. *P. ginseng* has also been used to treat CRC via autophagy induction [76]. *P. ginseng* extract upregulated the protein levels of Atg5, Beclin-1, and LC3 II, which are responsible for autophagosome recruitment in HCT-116 and SNU-1033 cells. This effect leads to lysosomal hydrolase of the cell organelles, causing cell death.

### 2.3. Reducing Cancer Cachexia

Cancer cachexia, which can result from long-term use of chemotherapy, accompanies body weight loss and inflammation. Surprisingly, a purified *P. ginseng* extract called BST204, containing 12.1% Rg3 and 7.1% Rh2, significantly reduced the symptoms of cancer cachexia induced by intraperitoneal injection of 5-FU [80]. Specifically, 200 mg/kg of BST204 increased the tumor-excluded body weight of 5-FU-treated BALB/c mice inoculated with 1 × 10^6^ CT26 CRC cells. The muscle weight and fibers also recovered to the basal level. The effect was driven by the anti-inflammatory and antioxidant properties of BST204, which alleviates the pro-inflammatory IL-6 serum level in mice.

### 2.4. Microbiota Population

As the colon environment is significantly affected by gut microbiota, natural products that change the population of gut microbiota have been extensively studied [81,82]. Indeed, *P. notoginseng* is one of the main modulators of gut microbiota [83,84]. For instance, oral injection of *P. notoginseng* root extract (30 and 90 mg/kg) strengthens the effect of an immunomodulatory bacterium called *Akkermansia muciniphila* via triggering the synthesis of gut microbial metabolite CK. This results in the activation of a T-cell-mediated immune response [85]. Thus, the extract injection eventually ameliorated the colon tumor production in male A/J mice induced by azoxymethane and dextran sodium sulfate (DSS) [86].

Overall, extract derived from the genus *Panax* can kill CRC cells by inducing apoptosis, reducing the side effects of chemotherapy by alleviating cancer cachexia, and suppressing tumor growth via a modulatory effect on gut microbiota (Table 2).

## 3. Study on the Mechanism of Colon Cancer Treatment of Ginsenosides and Their Metabolites

Ginsenosides are triterpenoid saponins isolated from various types of ginseng such as *P. ginseng*, *P. notoginseng*, and *P. quinquefolium* [87,88]. More than 150 ginsenosides have been found [87,88,89]. Most ginsenosides consist of 17 carbons in a four-ring structure, called a dammarane skeleton, with different sugar parts [90]. Dammarane-type saponins are categorized into several groups. When dammarenediol-II, a precursor of saponin, is hydroxylated, PPD is formed [91]. Then, PPD is *O*-glycosylated to the final saponins [21]. Ginsenosides Rb1, Rb2, Rc, Rd, and compound K are PPD-type saponins [92]. PPD can be hydroxylated again to PPT. PPT-type saponins Re and Rg1 are synthesized via the *O*-glycosylation of PPT [21]. The structure of ginsenosides depends on different hydroxyl groups and sugar groups on the C-3 and C-6 positions in the skeleton [93]. The structures are highly related to the bioactivities of ginsenosides, including anti-cancer, anti-inflammatory, and neuroprotective effects [94]. In particular, it has been reported that ginsenosides have efficient anti-cancer activity in a variety of cancers, such as gastrointestinal cancer, lung adenocarcinoma, breast cancer, prostate cancer, and small-cell lung cancer [46,95,96,97,98,99].

Cancer cell proliferation can be blocked by arresting the cell cycle [100]. Ginsenosides inhibit cancer cell proliferation in several cancers by targeting the cell cycle [43,44,101]. The anti-cancer effect can be proven by suppressing invasion and metastasis, which induce the progression of cancers [102]. The expression of MMP is a marker of cancer invasion and metastasis [103]. Specific ginsenosides reduce the expression of MMP-2 and MMP-9, preventing invasion of cancer cells [46,104]. The tumor volume and metastasis level of a tumor can also determine the efficacy of medicines [105]. In addition, it was reported that some ginsenosides resolve multi-drug resistance (MDR) of cancer chemotherapy [101]. Ginsenosides regulate these anti-cancer effects with different mechanisms. Ginsenosides induce the apoptosis of cancer cells by increasing pro-apoptotic proteins and decreasing anti-apoptotic proteins [106,107]. Moreover, it was reported that ginsenosides regulate PI3K/AKT signaling, inhibiting cancer growth and leading to therapeutic effects [108,109]. MAPK signaling, which is known for its cancer-promoting effect, is also a target mechanism of ginsenosides [110,111].

### 3.1. Rh2

#### 3.1.1. Anti-Inflammatory Activity

Ginsenoside Rh2 is one of the saponins extracted from ginseng. Rh2 has been researched as an appropriate aspect of cancer therapy due to its anti-proliferation, anti-invasion, anti-metastasis, and even anti-MDR effects [112]. Rh2 controls cancer properties by regulating miRNAs [113,114]. Chen et al. showed that Rh2 decreased the expression level of STAT3 and miR-214 level by downregulating the expression of pro-inflammatory cytokine IL-6, leading to an increase in PTEN [113]. Rh2 (50 mg/kg/d) also reduced the expression of other cytokines, IL-1*β* and TNF-*α*. Suppression of the miR-214 level alleviated inflammation of colitis, repairing the length of intestines and intestinal mucosa.

#### 3.1.2. Arresting Cell Cycle and Apoptosis

On the other hand, Rh2 (10 and 20 μM) increased the level of miR-150-3p, which was downregulated in colon cancer [114]. Then, upregulated miR-150-3p reduced SRCIN1 and inactivated the Wnt pathway via the miR-150-3p/SRCIN1/Wnt axis. Rh2 also induced the apoptosis of CRC cells by increasing Bax and caspase-3 and decreasing proliferating cell nuclear antigen (PCNA), cyclin D1, Myc, and β-catenin. In addition, colony formation, migration, and invasion of CRC cells were suppressed by Rh2 treatment.

Ginsenoside Rh2 showed a synergistic effect with other cancer therapies [115,116]. Ma et al. suggested that Rh2 resolved OX resistance in CRC [115]. Rh2 upregulated the expression of Smad4 and downregulated the expression of P-gp in L-OHP-resistant Lovo CRC cells. Apoptosis was also induced by Rh2 treatment, leading to increases in Bax and caspase-3 and a decrease in Bcl-2.

#### 3.1.3. Synergistic Therapy with Radiation

Lee et al. reported that Rh2 and radiation therapy demonstrated synergy in treating colon cancer both in vitro and in vivo [116]. Rh2 combined with 4-Gy radiation inhibited the activity of NF-κB, AKT, ERK, p38, and JNK. Rh2 suppressed MMP-9, VEGF, Bcl-2, cyclin D1, and PD-1 while increasing the expression of IL-12, IL-18, and IFN-*γ.* Rh2 (10 mg/kg) and radiation therapy induced cell apoptosis and decreased the tumor volume with an increase in the helper T cell/cytotoxic T cell population.

### 3.2. Rg3

The ginsenoside Rg3 possesses various biological activities, including not only hepato-protective and neuroprotective effects but also anti-cancer effects. It was reported that Rg3 regulates anti-proliferation, anti-metastasis, and anti-angiogenesis [117]. Hong et al. demonstrated that Rg3 enhanced the anti-cancer effect of 5-FU by increasing E-cadherin and Apaf-1 and decreasing N-cadherin and MMP-9, inhibiting colony formation, migration, and invasion [118]. Rg3 treatment also caused the apoptosis of colon cancer cells, leading to the upregulation of cleaved caspases 9 and 3 and downregulation of cyclin D1, CDK2, and CDK4. Rg3 showed an anti-cancer effect by suppressing the activities of p85, p110β, PDK1, and AKT in the PI3K/AKT signaling pathway. It also significantly reduced the tumor volume, diversity of gut microbiota, and weight in animal experiments. Furthermore, Rg3 induced mitophagy by increasing ubiquitinated GAPDH, followed by increases in Parkin and PINK1 in colon cancer cells [119]. Rg3-induced mitophagy was detected via high levels of LC3-Ⅱ and SQSTM1/p62 with autolysosomes, while Rg3 decreased the expression of VDAC1 and MFN2.

### 3.3. Rb1

The ginsenoside Rb1 is a major ginsenoside that inhibits the expression of pro-inflammatory cytokines such as IL-6 and TNF-*α* [29,120]. Wang et al. showed that AOM/DSS-induced CRC was alleviated by Rb1 treatment [121]. Rb1 regulated different cytokines, decreasing TNF-*α*, IL-6, IL-17A, IL-33, IL-1β, and IL-22 while increasing IL-10. Lu et al. also reported that Rb1 (10.72 mg/kg) successfully downregulated TNF-*α* and IL-6 levels in a cancer cachexia mouse model [122]. Moreover, the weight of the liver, which became heavy after the xenograft, decreased as a result of Rg3 treatment.

### 3.4. Secondary Metabolites

Ginsenosides are secondary metabolites synthesized from *P. ginseng* [28,123]. Ginsenosides are classified as dammarane, ocotillol (OCT), or oleanane (OA)-type saponins [28,92]. There are various species of ginsenoside of dammarane types, which are divided among PPD and PPT types, depending on the sugar moiety [92]. The sugar moiety is a key factor showing diverse biological activities [124]. Xiao et al. replaced the functional group of panaxadiol with Br, F, or Cl [125]. Some of these panaxadiol derivatives showed cell cytotoxic ability at low concentrations in colon cancer cells. On the other hand, Wang et al. suggested an anti-cancer effect of panaxadiol itself [126]. Panaxadiol targeted JAT/STAT signaling and MAPK signaling, inhibiting the activation of JAK1/2, Src, STAT3, ERK1/2, JNK, and p38. In addition, panaxadiol downregulated the phosphorylation of hypoxia-related proteins, HIF-α, 4EBP1, ElF4E, mTOR, and P70S6K, including cell death with decreases in cyclin D1, VEGF, and c-Myc. Moreover, the expression of PD-L1 was decreased, interfering with immune escape. Panaxadiol also increased LDH release and TNF-*α*/IFN-*γ* secretion in a co-culture with T lymphocytes and HCT-116 colon cancer cells. The cell viability, colony-forming ability, and tumor volume were also reduced by panaxadiol treatment. CK is one of the PPD-type ginsenoside metabolites which has pharmacological activities, including anti-cancer, antioxidant, and anti-proliferation [94,127]. Pak et al. demonstrated that CK caused the apoptosis of CRC cells via caspase 3 and p53-dependent LGR5 inhibition [128]. CK induced the sub G1 phase of colon cancer cells and decreased the expression of LG5, c-Myc, Pin1, pro-caspase 3, pro-PARP, Bcl-XL, Snail, and cyclin D1. However, there was no significant change in p53 KO cells, verifying that the apoptosis was mediated by p53.

Wu et al. reported that protopanaxatriol (20, 50, and 100 mg/kg) successfully controlled the metabolism of riboflavin, arachidonic acid, and glycerophospholipids [129]. Protopanaxatriol also showed a pharmacological effect in an animal model. It led to the recovery of the length of the intestine and size of the spleen and alleviated decreases in the body weight and inflammation level. Furthermore, the expression of cytokines, including IL-6, IL-1*β*, and TNF-*α*, was downregulated by protopanaxatriol.

To sum up, ginsenosides targeted CRC cells, both in vitro and in vivo levels, by regulating a wide range of molecular pathways, including PI3K/Akt and JAK/STAT, as well as the inactivation of pro-inflammatory cytokines such as IL-6 and TNF-α (Table 3).

## 4. Ginsenosides with or as a Drug Carrier

While ginsenosides show intrinsic efficacy, they also show potential as a versatile carrier for delivery purposes to treat CRC (Table 4). In the formulation of NPs or lipid nanocarriers, encapsulating ginsenosides can not only reduce toxicity and side effects but also enhance its therapeutic effects.

### 4.1. 20(S)-Ginsenoside Rg3-Based Polypeptide NPs

Polypeptide NPs can act as an all-round carrier for efficient delivery [130]. The functional group of amino acid facilitates the electrostatic interaction between Rg3 and NPs [131]. Furthermore, hydrophobic amino acid (e.g., phenylalanine) boosts the hydrophobic/aromatic interaction, resulting in the expanded uptake of drugs and connection with NPs [132]. These connections support the successful administration of drugs in the tumor tissues [133]. The tumor surroundings have an acidic condition [134], and polypeptide NPs can block the separation with ginsenosides in neutral pH conditions [135]. This means that these particles can maintain the self-assembly state in normal tissue and dissociation only in acidic tissue (e.g., tumors) [136]. Additionally, the biodistribution to tumor tissue and the pharmacokinetics for drug release are much better than those of free Rg3. Free Rg3 showed transition to the liver and kidney and boosted release [137]. In contrast, the Rg3-based polypeptide NPs mostly stacked to tumor tissue induced by the injection of SW480 CRC cells in mice, and the half-life was much longer than that of free Rg3 [138]. Moreover, Rg3 NPs only reduce the serum tumor factor called the carcinoembryonic antigen (CEA), where no other factors are related to normal tissue. This resulted in the efficient necrosis of tumor tissue. PCNA is related to tumor cell proliferation, and Caspase-3 is related to apoptosis. Rg3 NPs manifested the downregulation of PCNA and upregulation of Caspase-3 towards tumor death.

### 4.2. Ginsenoside-Modified Nanostructured Lipid Carriers Containing Curcumin

Curcumin is a type of polyphenol and the main active compound of turmeric plants [139]. It has well-known antioxidative and anti-inflammatory effects [140]. These effects can reduce the risk of cancer, cardiovascular disease, Alzheimer’s disease, and rheumatoid arthritis [141,142,143,144]. However, the bioavailability of curcumin is limited by a rapid metabolism process [145]. Therefore, assistance is required. Cholesterol and derivatives are necessary ingredients to stabilize the phospholipid [146]. This implies that the steroid structure of ginsenosides may improve the stabilization and upregulate absorption of curcumin [147]. Previous nano-lipid carriers exhibited imperfections like a low curative effect [148,149]. However, a novel ginsenoside-modified nanostructure lipid carrier containing curcumin showed better results of cytotoxicity, uptake level, and plasma level of CRC patients with unresectable tumors [150]. In addition, it strengthens the survival ratio when used with general drugs for unresectable metastatic CRC patients [151].

### 4.3. Three-Layer Functional Polymer Materials with Ginsenoside

Biodegradable polymer materials represent a hot research topic in nanomaterials. They are used in the treatment of many diseases, such as cancer [152,153]. The benefits of electrospun nanofibers include loading drugs, great stability, and suitable pharmacokinetics [154]. Additionally, biodegradable hydrogel is a valuable material due to its hydrophilicity, non-toxicity, and biocompatibility [155]. Because of these characteristics, a combination of two components can deliver many drugs effectively. In CRC, the interaction between sialic acid expressed on the surface of tumors [156] and PA-decorated PEG-PLGA polymer hydrogel enhances the accuracy of tumor targeting [157]. In addition, the structure of Rg3-combined hydrogel and 5-FU (a general chemotherapeutic cancer drug) gradually killed tumor cells. The combined three-layer particles killed tumors more effectively, as demonstrated by the lowest tumor volume, weight, and CEA concentration as well as the immunoglobulin cell adhesion molecule agitated in cancer patient’s serum [158,159]. In cancer cells treated with three-layer particles, Caspase-3 was upregulated, and bcl-2, Ki-67, and VEGF were downregulated, which is related to an increase in apoptosis and decrease in tumor cell growth. Moreover, it can reduce the toxicity in the liver and exhibit no effects on the heart and kidney. This means that this particle might be safer than other conventional drugs, which have side effects in major organs.

**Table 4:** Ginsenosides as drug carriers to target CRC. ↑ increased, ↓ decreased.

Nanocarriers	Ginsenosides	Advantages	Mechanisms	Ref.
mPEG-b-P(Glu-co-Phe) NP	20(S)-ginsenoside Rg3	Maintenance for NPs Dissociation in acidic conditions (e.g., tumors) Effective biodistribution Slow pharmacokinetics	PCNA ↓ Caspase-3 ↑	[138]
Nanostructured lipid carrier containing curcumin	Rg1, Rd, F2, protopanaxadiol, Rg3, compound K, protopanaxatriol	Bioavailability ↑ Lipid particle stabilization ↑ Cytotoxicity in tumors ↑ Uptake level ↑	ND	[150]
	Hydrolyzed ginsenoside	Survival rate ↑	ND	[151]
Polylactic acid polyglycolic acid	Rg3	Induction of apoptosis Tumor weight, size ↓	Caspase-3 ↑ CEA Bcl-2, Ki67, VEGF, ALT/AST, ↓	[159]

## 5. Conclusions

In this review, we summarized research focusing on the pharmacological role of the genus *Panax* and its active compounds—namely ginsenosides —in the context of CRC. The extracts from plants of the *genus Panax* downregulated CRC cells through multiple mechanisms, including apoptosis, inducing autophagy, alleviating cancer cachexia, and controlling the gut microbiota. *P. ginseng* and *P. quinquefolium* induced apoptosis following suppression of the PTEN/PI3K/Akt pathway and Bax activation and activated autophagy through increasing the expression of Atg5, Beclin-1, and LC3 II. *P. notoginseng* triggered the T-cell immune response by increasing the population of *Akkermansia muciniphila*.

Recent studies on the anti-cancer effects of ginsenosides mainly covered the effects of PPD-type ginsenosides, including Rh2, Rg3, and Rb1. Rh2 alleviated the inflammatory response, decreasing the pro-inflammatory cytokine level in both the CRC cell line and xenograft mouse model. This ameliorated colitis and induced the apoptosis of CRC cells. Rh2 controlled the interaction of miR-150-3p/SRCIN1 to block the colony formation, invasion, and migration of CRC cells. Rg3 regulated the PI3K/Akt-signaling cascade and Parkin-dependent mitophagy while inducing pro-apoptotic proteins in CRC cells. Meanwhile, Rb1 modulated the immune cell response via shifting the levels of pro-inflammatory cytokines, such as TNF-*α* and IL-6, in mice induced with AOM and DSS. Panaxadiol and its derivatives, including CK, also exerted anti-cancer activities on CRC through inducing cell cycle arrest, LDH release, and intrinsic apoptosis. Interestingly, PPT with an anti-inflammatory effect (e.g., suppression of IL-6, IL-1*β*, and TNF-*α*) ameliorated colitis, resulting in the recovery of body weight and colon length.

**Figure 3:**
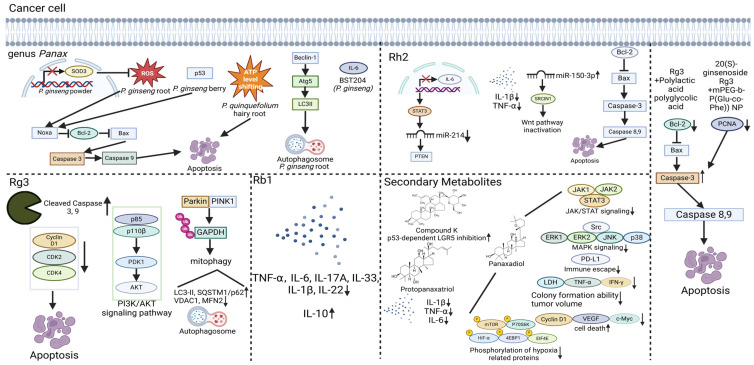
Summary illustration of ginseng and ginsenosides for CRC therapeutics. ↑ increased, ↓ decreased.

Encouragingly, there is growing emphasis on the development of biocompatible carriers for ginsenosides, such as an Rg3-loaded hydrogel using mPEG-b-PLGA polymers, in order to enhance delivery efficiency [138]. Nanocarriers supported the slow pharmacokinetics of Rg3, helping the compound to specifically target tumor regions. Moreover, ginsenosides themselves are gaining attention as novel drug delivery vehicles [150,151]. Various ginsenosides, including Rg1, Rd, and Rg3, acted as a key component of nanostructured lipid carriers, which move curcumin to tumor lesions. This enhanced the cytotoxic effect of curcumin against CRC cells by stabilizing the lipid particle. A Rg3-combined biodegradable polymer coated with three layers helped the activity of 5-FU, upregulating the death of CRC cells in patients. The three-layer structure supplemented with Rg3 safely transports 5-FU to cancerous tissues without expressing toxicity in the heart and kidneys [159]. These studies suggest that ginsenosides can be used to treat CRC in the human body, as illustrated in Figure 3.

In conclusion, we hope that more researchers will be motivated by this review to conduct experiments and develop commercialized drugs or drug formulations that can successfully cure CRC.

## Figures and Tables

**Table 1:** The structure of PPD-type ginsenosides with their R groups.

Ginsenoside	Type	(C-3) R^1^	(C-20) R^3^
Rh2	PPD type	Glc	H
Rg3		Glc(2 → 1)Glc	H
Rb1		Glc(2 → 1)Glc	Glc(6 → 1)Glc
CK		H	Glc
PPD		H	H

H: hydrogen, Glc: glucose.

**Table 2:** Pharmaceutical effect of genus *Panax* on CRC.

Extract	Test Type	Dose	Mechanism	Reference
*P. ginseng* powder	In vitro (HT-29 CRC cell line)	2.5 mg/mL (for 24 h)	- Extract downregulates the transcription of SOD3 - Decreased SOD3 activates ROS-induced Noxa activation - Noxa induces an intrinsic apoptotic pathway	[74]
*P. ginseng* berry	In vitro (HCT-116 and HT-29 CRC cell lines) Ex vivo (naïve CD4 cells from C57BL/6 mice spleen)	100, 200, 300, 400, and 500 μg/mL (for 48 h)	- Extract induces an intrinsic apoptotic pathway via p53 activation - Extract induces cell cycle arrest via cyclin A activation - Extract inhibits the differentiation of Th17 cells	[75]
*P. ginseng* root	In vitro (HCT-116 and SNU-1033 CRC cell lines)	2 mg/mL for HCT-116 cells, 2.3 mg/mL for SNU-1033 cells (for 12, 24, and 48 h)	- Extract induces oxidative stress in CRC cells, upregulating the intrinsic apoptotic pathway - Extract increases the protein level of Atg5, Beclin-1, and LC3 II, which results in autophagy activation	[76]
*P. quinquefolium* hairy root (elicited by methyl jasmonate)	In vitro (Caco-2 CRC cell line)	0.017, 0.069, 0.274, 0.55, and 1.1 mg/mL (for 48 h)	- Extract blocks the proliferation and colony formation of CRC cells - Extract treatment results in the decrease in ATP level, shifting the mitochondrial membrane potential and triggering apoptosis	[77]
BST204 (*P. ginseng)*	In vivo (BALB/c xenograft mouse model with 1 × 10^6^ of CT-26 CRC cell line treated with 50 mg/kg of 5-FU)	100 and 200 mg/kg (5 d cycles for 11 d)	- Extract recovers the tumor-excluded body weight - Extract increases the muscle and fat weight, which was lowered by 5-FU injection - Extract administration reduces the serum IL-6 level - Extract regulates the balance in protein degradation and stabilization as well as glucose metabolism	[80]
*P. notoginseng* root	In vivo (A/J mouse CRC model with 7.5 mg/kg of azoxymethane and 1% DSS)	30 and 90 mg/kg (for 13 weeks)	- Extract recovers the colon length and suppresses tumor development in the colon - Extract shifts gut microbiota population by increasing the population of *A. muciniphila*	[86]

**Table 3:** Pharmaceutical effects of ginsenosides on CRC.

Ginsenoside	Test Type	Dose	Mechanism	Reference
Rh2	In vitro (NCM460 CRC cell line) In vivo (C57BL/6J CRC mouse model with 3% DSS-induced acute colitis)	1.25, 2.5, 5, 10, and 20 μM (for 24 h) 50 mg/kg/day (for 10 days)	- Rh2 decreased STAT3 and miR-214 levels and increased PTEN by suppressing IL-6 expression - Rh2 decreased IL-1*β* and TNF-*α* - Rh2 alleviated colitis	[113]
	In vitro (HCT-116, SW620 CRC cell line)	10 and 20 μM (for 18 h)	- Rh2 increased miR-150-3p - Rh2 decreased SRCIN1 and inhibited Wnt signaling - Rh2 induced apoptosis and suppressed colony formation, invasion, and migration of CRC cells	[114]
	In vitro (Lovo, Lovo/L-OHP CRC cell line)	50, 100, 200, and 250 μg/mL (for 24 h)	- Rh2 increased Smad4 and decreased P-gp in OX-resistant CRC cells - Rh2 induced the apoptosis of OX-resistant CRC cells	[115]
	In vitro (CT26/luc CRC cell line) In vivo (BALB/c xenograft mouse model with 2 × 10^6^ CT-26 CRC cells)	1, 20, 50, 75, 85, and 100 μM (for 24 h) 10 mg/kg (for 3 weeks, three times a week)	- Rh2 combined with radiation inhibited NF-κB, AKT, ERK, p38, and JNK activation - Rh2 decreased MMP-9 and VEGF - Rh2 induced the apoptosis of CRC cells - Rh2 increased the helper T cell/cytotoxic T cell population and reduced tumor volume	[116]
Rg3	In vitro (SW620, Lovo CRC cell line) In vivo (nude mice xenograft mouse model with 5 × 10^6^ human CRC cells)	0.25, 0.5, 0.75, and 1.0 mmol/L (for 48 h) 200 mg/kg (for 3 weeks)	- Rg3 targeted PI3K/AKT signaling and inhibited tumors - Rg3 increased E-cadherin and Apaf1 and decreased N-cadherin and MMP-9 - Rg3 increased pro-apoptotic proteins and decreased anti-apoptotic proteins to induce apoptosis	[118]
	In vitro (HCT-116 CRC cell line)	20 μM (for 12 h)	- Rg3 regulated Parkin and PINK1 expression and increased ubiquitinated GAPDH - Rg3 induced Parkin-dependent mitophagy and decreased the expression of VDAC1 and MKN1	[95]
Rb1	In vivo (C57BL/6 CRC mouse model with single dose of 7.5 mg/kg AOM and 2% DSS for 7 days)	No information (18 weeks, every 2 days)	- Rb1 decreased the expression of TNF-*α*, IL-6, IL-17A, IL-33, IL-1β, and IL-22, while increasing the expression of IL-10 - Rb1 alleviated tumors and pathology and increased diversity of microbiota	[121]
	In vivo (BALB/c xenograft mouse model with 1 × 10^6^ CT-26 CRC cells)	10.72 mg/kg (for 23 days)	- Rb1 decreased the expression of TNF-*α*, and IL-6 - Rb1 reduced the weight of the liver	[122]
Panaxadiol derivatives	In vitro (HCT-116 CRC cell line)	0.5–2 mM	- Panaxadiol derivatives showed cell cytotoxicity ability at low concentrations	[125]
Panaxadiol	In vitro (HCT-116, SW620, HT-29 CRC cell lines) In vivo (BALB/c nude xenograft mouse model with 5 × 10^7^ HCT-116 CRC cells)	1, 3, and 10 μM (for 24 and 48 h) 10 and 30 mg/kg (36 days, three times a week)	- Panaxadiol decreased the phosphorylation of JAK1/2, Src, STAT3, ERK1/2, JNK, and p38 - Panaxadiol decreased the phosphorylation of HIF-α, 4EBP1, ElF4E, mTOR, and P70S6K - Panaxadiol decreased the expression of cyclin D1, VEGF, and c-Myc and included cell death - Panaxadiol decreased the expression of PD-L1 and interfered with immune escape - Panaxadiol increased LDH release and TNF-*α*/IFN-*γ* secretion in co-culture (T lymphocytes and HCT-116 colon cancer) cells - Panaxadiol decreased colony formation ability and tumor volume	[126]
Compound K	In vitro (HCT-116 CRC cell line)	6.25, 12,5, 25, 50, and 100 μM (24, 48 h weeks)	- Compound K caused apoptosis by decreasing the expression of LG5, c-Myc, Pin1, pro-caspase 3, pro-PARP, Bcl-XL, Snail, and cyclin D1 - Compound K arrested cell cycle to the sub G1 phase in a p53-dependentmanner	[128]
Protopanaxatriol	In vivo (BALB/c CRC mouse model with 3% DSS-induced acute colitis)	25, 50, and 100 mg/kg (7 days)	- Protopanaxatriol controlled the metabolism of riboflavin, arachidonic acid, and glycerophospholipids - Protopanaxatriol restored the length of the intestine and size of spleen, and alleviated the decrease in body weight and inflammation level - Protopanaxatriol decreased the cytokine expression of IL-6, IL-1*β*, and TNF-*α*	[129]

## Data Availability

The data are contained within the article.

## Abbreviations

*CRC*	*Colorectal cancer*
*PPD*	Protopanaxadiol
*P. notoginseng*	Panax notoginseng
CIN	Chromosomal instability
MSI	Microsatellite instability
5-FU	Fluoropyrimidine
OX	Oxaliplatin
IRI	Irinotecan
FOLFOX	5-FU + folinic acid + OX
FOLFIRI	5-FU + folinic acid + IRI
FOLFOXIRI	5-FU + folinic acid + OX + IRI
*P. quinquefolium*	*Panax quinquefolium*
*P. ginseng*	*Panax ginseng*
PPT	Protopanaxatriol
CK	Compound K
NPs	Nanoparticles
Bcl-2	B-cell lymphoma-2
Bax	Bcl-2-associated X protein
PCNA	Proliferating cell nuclear antigen
CEA	Carcinoembryonic antigen
ER	Endoplasmic reticulum
